# Genetic risk model for in-stent restenosis of second-and third-generation drug-eluting stents

**DOI:** 10.1016/j.isci.2021.103082

**Published:** 2021-09-03

**Authors:** Yen-Wen Liu, Mu-Shiang Huang, Ling-Wei Hsu, Hsien-Yuan Chang, Cheng-Han Lee, Chi-Ying Lee, Dao-Peng Chen, Yi-Heng Li, Ting-Hsin Chao, Pei-Fang Su, Meng-Ru Shen, Ping-Yen Liu

**Affiliations:** 1 Division of Cardiology, Department of Internal Medicine, National Cheng Kung University Hospital, College of Medicine, National Cheng Kung University, Tainan 704, Taiwan; 2 Institute of Clinical Medicine, College of Medicine, National Cheng Kung University, 138 Sheng-Li Road North District, Tainan 704, Taiwan; 3 KimForest Enterprise Co., Ltd., New Taipei City 221, Taiwan; 4 Department of Statistics, National Cheng Kung University, Tainan 704, Taiwan; 5 Department of Obstetrics and Gynecology, National Cheng Kung University Hospital, College of Medicine, National Cheng Kung University, Tainan 704, Taiwan; 6 Department of Pharmacology, College of Medicine, National Cheng Kung University, Tainan 704, Taiwan

**Keywords:** Medicine, Clinical genetics, Drugs

## Abstract

The new generation, i.e., second- and third-generation, drug-eluting stents (DESs) remain a risk of in-stent restenosis (ISR). We evaluated the power of a genetic risk score (GRS) model to identify high-risk populations for new generation DES ISR. We enrolled patients with coronary artery disease (CAD) treated with new generations DESs by a single-center cohort study in Taiwan and evaluated their genetic profile. After propensity score matching, there were 343 patients and 153 patients in the derivation and validation cohorts, respectively. Five selected single-nucleotide polymorphisms (SNPs), i.e., SNPs in *CAMLG*, *GALNT2*, *C11orf84*, *THOC5*, and S*AMD11*, were included to calculate the GRS for new generation DES ISR. In the derivation and the validation cohorts, patients with a GRS greater than or equal to 3 had significantly higher new generation DES ISR rates. We provide biological information for interventional cardiologists prior to percutaneous coronary intervention by specific five SNP-derived GRS.

## Highlights

• A validated GRS model identified high-risk population for new generation DES ISR
• This GRS includes 5 SNPs in exons: *CAMLG*, *GALNT2*, *C11orf84*, *THOC5*, and S*AMD11*
• The patients with high GRSs (≥3) had higher rates of new generation DES ISR
• The GRS provides crucial information in shared decision-making process clinically

## Graphical abstract

**Figure:**
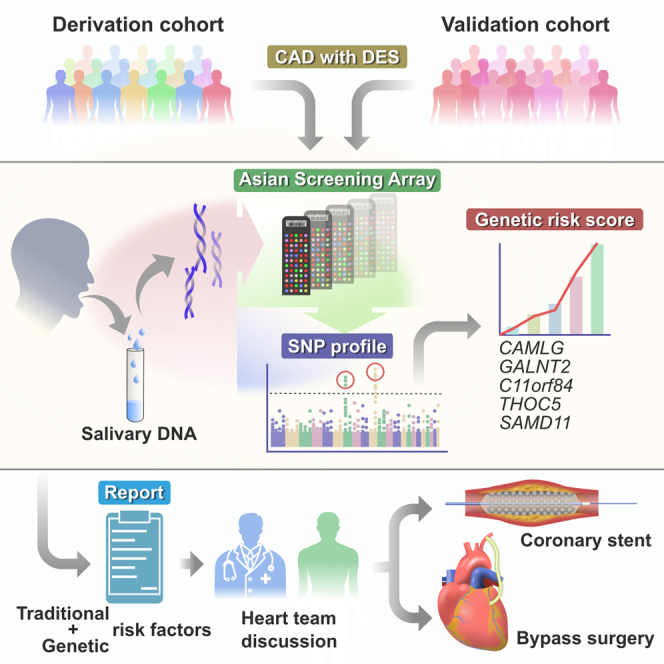

## Introduction

Coronary artery disease (CAD) has been the leading cause of mortality worldwide for decades. CAD is characterized by reduced blood supply to the myocardium, which can lead to ischemia and even myocardial infarction. Because coronary artery stenosis is the most common cause of CAD, percutaneous coronary intervention (PCI) for angioplasty of the narrowing lesions has become a well-established therapeutic practice. Compared with balloon angioplasty and bare metal stent (BMS) deployment, drug-eluting stents (DESs) significantly reduce the incidence of restenosis and improve patient prognosis (Bonaa et al., 2016). Owing to improvements in stent design, drugs and polymers of DESs, and application of intracoronary imaging modalities during PCI, the in-stent restenosis (ISR) rate of the new generation DES, i.e., second- and third-generation DESs, is very low (Bonaa et al., 2016; Shlofmitz et al., 2019; Piccolo et al., 2015). Despite this, new generation DES ISR remains a clinical challenge.

There are several possible pathophysiological mechanisms to explain the etiology of new generation DES ISR, including mechanical factors, technical issues, and biological aspects (Dangas et al., 2010; Shlofmitz et al., 2019). According to the proposed pathogenesis of DES ISR, many strategies, such as application of intracoronary imaging during PCI and redesigned DES structures and coating drugs, have been applied to reduce new generation DES ISR rates. Compared with BMS and first-generation DESs, these efforts have significantly reduced ISR rates of new generation DESs, although biological issues, including genetic variants, have still not been overcome (Cutlip et al., 2002; Kimura et al., 2012; Raber et al., 2011; Piccolo et al., 2015).

In the last decade, many genome-wide association studies (GWASs) have been conducted to investigate the associations between genetic variants and cardiovascular diseases (Kathiresan et al., 2008; Mega et al., 2015; Musunuru and Kathiresan, 2019; Ntalla et al., 2019). Owing to significant advances in bioinformatics and big data analysis, GWAS-derived genetic risk scores (GRSs) have been applied for risk stratification of cardiovascular diseases (Khera et al., 2016; Natarajan et al., 2017; Inouye et al., 2018; Mega et al., 2015; Hajek et al., 2018; Ntalla et al., 2019). However, most of these studies were conducted in European populations (Levin and Rader, 2020; Verschuren et al., 2012). Because of the major impact of ethnic genetic variability, these genetic study findings cannot be applied to East Asian populations. Additionally, to date, no genetic studies have investigated new generation DES ISR.

Therefore, in this study, we aimed to identify high-risk populations for new generation DES ISR in East Asians and to evaluate the power of GRSs to detect new generation DES ISR in the Taiwanese population.

## Results

### Algorithm for the GRS of DES-ISR model

Through December 2019, there were 2,749 patients receiving new generation DES deployment in the NCKUH CAD cohort. Among these 2,749 patients, 205 patients had DES ISR, which was confirmed by coronary angiography. However, after screening, only 690 patients with CAD with new generation DES deployment were enrolled for genomic research, and 92 patients had DES ISR (Figure S1). We applied quality control filters to check the quality of extracted DNA from the enrolled patients’ saliva, and the sample call rate for GWAS analysis was more than 97%. The median follow-up duration in this cohort was 2.75 years (25^th^ and 75^th^ percentiles: 1.42 and 4.71 years, respectively). Furthermore, the median duration between DES deployment and ISR was 1.54 years (25^th^ and 75^th^ percentiles: 0.65 and 2.95 years, respectively).

After excluding patients with missing clinical information (n = 6) and CKD 5D (n = 54), 630 patients (age: 64.4 ± 10.1 years, male: 80%) were recruited for GRS analysis. These patients were categorized into two cohorts: derivation cohort (DES deployment between January 2010 and December 2017) and validation cohort (DES deployment between January 2018 and December 2019). However, because there were many significantly different variables between patients with and without DES ISR in these two cohorts, we performed propensity score matching to avoid confounding factors of DES ISR. Finally, there were 343 and 153 patients in the derivation and validation cohorts, respectively (Figure S1). There were no significant differences between patients with and without DES ISR in both the derivation cohort and the validation cohort (Table 1).

**Table 1:** Baseline clinical demographics of the derivation cohort and the validation cohort

CAD patients	Derivation cohort			Validation cohort		
	DES ISR (−) (n = 295)	DES ISR (+) (n = 48)	*p*	DES ISR (−) (n = 129)	DES ISR (+) (n = 24)	*p*
Age (years)	62.8 ± 9.9	60.5 ± 10.8	0.17	66.4 ± 10.5	65.4 ± 9.3	0.64
Male, n (%)	242 (82%)	37 (77.1%)	0.43	105 (81.4%)	20 (83.3%)	>0.99
BMI (kg/m^2^)	26.1 ± 4.0	25.5 ± 3.3	0.30	25.8 ± 4.0	25.5 ± 3.0	0.64
**Comorbidities**						
Diabetes mellitus, n (%)	143 (48.5%)	28 (58.3%)	0.22	45 (34.9%)	10 (41.7%)	0.64
Dyslipidemia, n (%)	280 (94.9%)	44 (91.7%)	0.32	116 (89.9%)	20 (83.3%)	0.31
Hypertension, n (%)	203 (68.8%)	35 (72.9%)	0.62	90 (69.8%)	14 (58.3%)	0.34
Old stroke, n (%)	12 (4.1%)	3 (6.2%)	0.45	15 (11.6%)	0 (0%)	0.13
CAD, n (%)	282 (95.6%)	46 (95.8%)	>0.99	121 (93.8%)	22 (91.7%)	0.66
CKD, n (%)	52 (17.6%)	11 (22.9%)	0.42	34 (26.4%)	6 (25%)	>0.99
Atrial fibrillation, n (%)	22 (7.5%)	5 (10.4%)	0.56	17 (13.2%)	3 (12.5%)	>0.99
Old MI, n (%)	122 (41.4%)	25 (52.1%)	0.21	59 (45.7%)	14 (58.3%)	0.28
Heart failure, n (%)	45 (15.3%)	10 (20.8%)	0.39	22 (17.1%)	6 (25%)	0.39
PAOD, n (%)	5 (1.7%)	2 (4.2%)	0.25	6 (4.7%)	1 (4.2%)	>0.99
**Medication**						
Aspirin, n (%)	252 (85.4%)	39 (81.2%)	0.51	109 (84.5%)	20 (83.3%)	>0.99
Clopidogrel, n (%)	216 (73.2%)	31 (64.6%)	0.23	88 (68.2%)	17 (70.8%)	>0.99
Ticagrelor, n (%)	50 (16.9%)	8 (16.7%)	>0.99	30 (23.3%)	7 (29.2%)	0.61
Factor Xa inhibitor, n (%)	5 (1.7%)	2 (4.2%)	0.25	2 (1.6%)	0 (0%)	>0.99
Factor II inhibitors, n (%)	1 (0.3%)	0 (0%)	>0.99	7 (5.4%)	2 (8.3%)	0.36
Warfarin, n(%)	4 (1.4%)	1 (2.1%)	0.53	4 (3.1%)	1 (4.2%)	0.59
ACEi/ARB, n(%)	149 (50.5%)	23 (47.9%)	0.76	49 (38%)	13 (54.2%)	0.16
β-blocker, n (%)	102 (34.6%)	20 (41.7%)	0.42	50 (38.8%)	8 (33.3%)	0.66
DHP-CCB, n (%)	34 (11.5%)	7 (14.6%)	0.63	18 (14%)	5 (20.8%)	0.36
Statin, n (%)	201 (68.1%)	26 (54.2%)	0.07	89 (69%)	16 (66.7%)	0.81

### GWAS analysis and the DES ISR-GRS model

**Figure 1:**
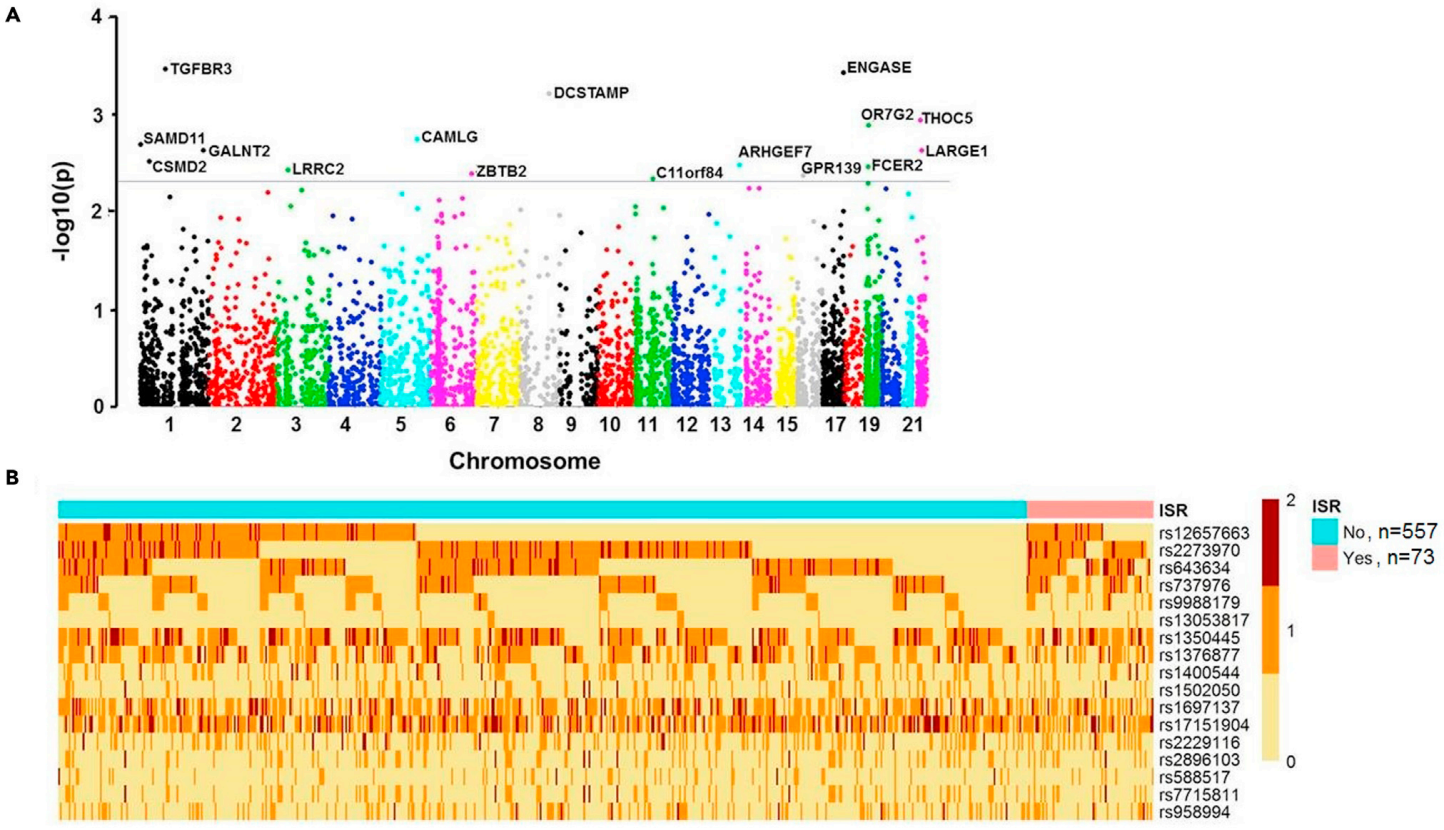
Comparison of single-nucleotide polymorphism (SNP) between the patients with and without new generation drug-eluting stent (DES) in-stent restenosis (ISR) (A) Manhattan plot for the genome-wide association study of exon SNPs in patients with new generation DES ISR. The definition of significant association was p < 0.005. (B) Heatmap analysis of the exonic SNP allele expression profile in patients with DES ISR and those without DES ISR. The SNP pools of patients with DES ISR (red) and without DES ISR (blue) are shown in columns. Rows demonstrate the expression of the candidate SNPs’ allele. The expressed SNP allele number, i.e. 0, 1, and 2, is indicated by yellow, orange, and brown colors, respectively.

We collected saliva samples from the recruited patients and extracted DNA for GWAS analysis. Our analysis focused on genetic variants in the exons and 3′-untranslated regions. There were 17 exonic single-nucleotide polymorphisms (SNPs) that met the inclusion criteria (Figure 1 and Table S1). To select high-impact SNPs, two SNPs with polyphen2 HDIV scores greater than 0.446 and three SNPs correlated with arteries in GTEx were included to construct the DES ISR-GRS model (Table 2) (Adzhubei et al., 2010; Consortium et al., 2017; Roselli et al., 2018).

**Table 2:** Details of exonic SNP of new generation drug-eluting stent in-stent restenosis genetic risk score model

Gene	RSID^TM^	*p*	Other Allele	Risk Allele	RAF	AA change	PolyPhen2 HDIV score	GTEx V6 tissue
CAMLG	rs12657663	0.0018583	C	T	0.22	V78I	NA	Coronary artery
GALNT2	rs2273970	0.0023998	G	A	0.34	V516M	0.764	NA
C11orf84	rs643634	0.0046966	A	C	0.32	NA	NA	Artery
THOC5	rs737976	0.0011858	T	C	0.23	V525I	NA	Artery
SAMD11	rs9988179	0.0020945	G	A	0.11	H78Y	0.989	NA

RSID^TM^, rapid stain identification series; RAF, risk allele frequency; GTEx, genotype-tissue expression; N/A, non applicable.

**Figure 2:**
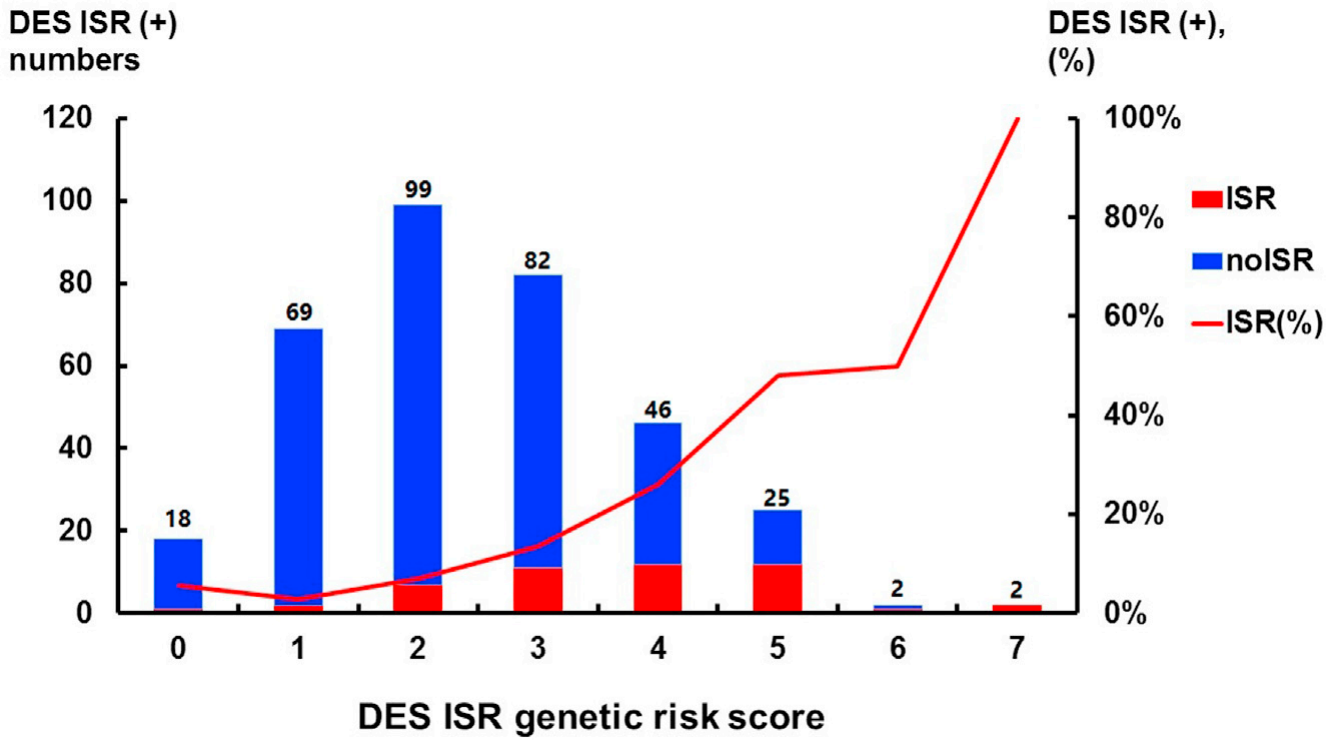
The distribution of new generation drug-eluting stent (DES) in-stent restenosis (ISR) patient numbers and percentage in different DES ISR genetic risk score

According to the definition of GRS calculation, the values for new generation DES ISR-GRS ranged from 0 to 10, but no patients had GRSs greater than or equal to 8. Thus, in our patients in the derivation cohort, the range of GRS values was between 0 and 7 (Table S2 and Figure 2). Furthermore, we found that patients with GRSs greater than or equal to 3 had significantly higher DES ISR rates (Figure 2). To determine the most optimal and reasonable cut-off value for DES ISR-GRSs, we performed diagnostic accuracy tests. When the cut-off value of the GRS was greater than or equal to 2, the sensitivity of the GRS associated with DES ISR was up to 93.8%, but its specificity and accuracy were low (28.5% and 37.6%, respectively). In contrast, when the GRS cut-off value was 4, the specificity and accuracy were higher, but the sensitivity was close to 50% (Table S1). As a result, we set the cut-off value of DES ISR-GRS as greater than or equal to 3.

### Prediction for the DES-ISR by different GRS scores

**Figure 3:**
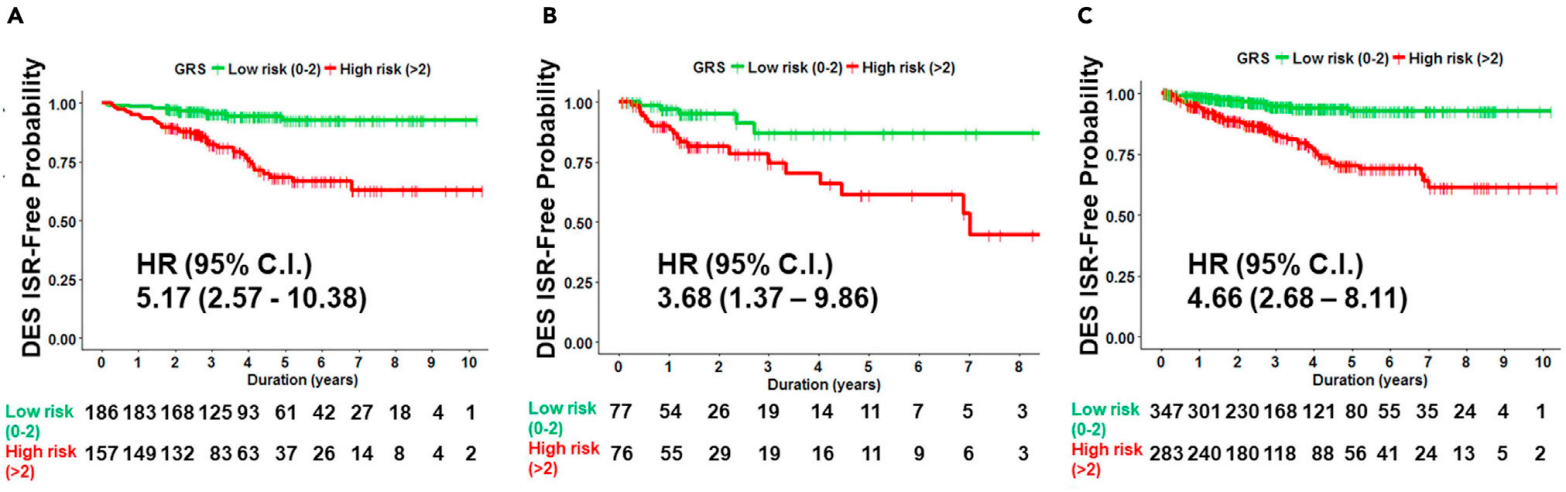
The Kaplan-Meier curve for derivation cohort, validation cohort, and total population of new generation drug-eluting stent (DES) in-stent restenosis (ISR) in patients with low (green) and high (red) genetic risk score (GRS) (A) derivation cohort. (B and C) Validation cohort and (C) total population. (A–C) The low GRS group (0–2) is shown in green color. The high GRS group (A–C) had higher incidence of DES ISR than the low GRS group did.

We estimated the effects of the GRS on DES ISR prevalence in the derivation cohort. The prevalence of DES ISR in patients with a DES ISR-GRS greater than or equal to 3 was significantly higher than that in patients with a GRS less than 3 (HR: 5.17, 95% CI: 2.57–10.38, p < 0.001, Figure 3A).

### Validation of the DES ISR-DRS model

Because the prevalence of new generation DES ISR is not high, it may be difficult to perform external validation in a large DES ISR cohort. Thus, we prospectively enrolled patients with or without DES ISR at our hospital from January 2018 to December 2019 for internal validation. We performed propensity score matching between DES ISR (+) patients and DES ISR (−) patients in this validation cohort. There were no significant differences between the two groups in this validation cohort (Table 1). The prevalence of DES ISR in the patients with a GRS greater than or equal to 3 was also significantly higher than that in patients with a GRS less than 3 (HR: 3.68, 95% CI: 1.37–9.86, p < 0.001, Figure 3B).

Finally, we checked the association between this DES ISR-GRS model and the CAD cohort receiving new generation DES deployment. We confirmed that patients with CAD having GRSs greater than or equal to 3 had a higher risk of DES ISR (HR: 4.66, 95% CI: 2.68–8.11, p < 0.001, Figure 3C).

## Discussion

In this study, we demonstrated, for the first time, that a five-SNP-derived GRS model, consisting of the *CAMLG*, *GALNT2*, *C11orf84*, *THOC5*, and S*AMD11* genotypes, was significantly associated with new generation DES ISR. Patients with CAD having high GRSs (e.g., ≥3) in both the derivation and validation cohorts had obviously higher event rates of new generation DES ISR. Thus, this GRS model may predict the risk of DES ISR events, as shown in our validation cohort, in which the patients were prospectively enrolled.

Compared with BMSs, DESs dramatically reduce the rate of major adverse events. Owing to improvements in PCI techniques, stent design, thin-struct alloy composition, coatings of polymers and drugs, and intravascular imaging application during PCI, the performance of new generation DESs is superior to that of first-generation DESs (Piccolo et al., 2015; Shlofmitz et al., 2019). Nevertheless, DES ISR was not completely eliminated, and the event rate was still approximately 6–15% (Piccolo et al., 2015; Cutlip et al., 2004; Dangas et al., 2010; Raungaard et al., 2015). Many mechanisms of new generation DES ISR have been proposed, and there are adaptive strategies to managing the pathogenesis of new generation DES ISR. Although genetic risk has not been clearly defined, it is known to play important roles in DES ISR. Some studies have revealed that genetic mutations can lead to resistance or insensitivity to coating drugs and may have a negative effect on the efficacy of DESs (Yusuf et al., 2003; Huang and Houghton, 2001; Verschuren et al., 2012); however, none of these studies investigated the influence of SNPs on new generation DES ISR. Furthermore, Hoppmann et al. (2014) reported that SNPs are risk factors for BMS ISR but are not helpful for risk stratification of DES ISR.

In this study, we identified candidate SNPs from the exons and 3′-untranslated regions having SNP call rates greater than 0.97, SNP AFs greater than 0.1, and p values less than 0.005. Then, according to the criteria of gene expression in the cardiovascular tissue or possible functional impact of risk variants (defined as Poly-Phen 2 HDIV score >0.446), five SNPs were evaluated using our DES ISR-GRS model. None of these candidate SNPs were tested by functional studies to confirm their biological impact on DES ISR, and no data for these five SNPs described the possible mechanisms leading to DES ISR; nevertheless, patients with high GRSs (defined as ≥ 3) had a higher incidence of DES ISR. Therefore, this DES ISR-GRS model could be used to identify patients at high risk of DES ISR in advance.

Regarding clinical applications, this DES ISR-GRS model could be applied for risk stratification of DES ISR and may be helpful for shared decision-making by patients, their families, and cardiologists/physicians with regard to coronary revascularization therapy. Additionally, if PCI is to be performed in patients having high DES ISR-GRSs, optimal PCI strategies, such as application of intravascular imaging and debulking techniques, should be used to reduce the risk of DES ISR, particularly for patients with high ISR GRSs.

In this study, those candidate SNPs have reported their roles in vascular diseases, leading to a possible mechanistic insight (Figure S2). CAMLG is an important signal transducer for the actions of angiotensin II in regulating the calcineurin-NFAT pathway. It is indicated that the interaction of CAMLG with angiotensin II type I receptor-associated protein (ATRAP) may mediate the angiotensin II actions in vascular physiology. ATRAP-interacting domain of CAMLG (aa 1-189) sensitized NFAT activation in response to angiotensin II (Guo et al., 2005; Min et al., 2009; Wakui et al., 2010). Our study demonstrated that CAMLG p.Val78Ile (rs12657663) is related to ISR and in Genotype-Tissue Expression (GTEx) Project risk allele T has higher mRNA expression. Spindlin1 (SPIN1) has been shown as a transcriptional coactivator of Wnt signaling (Devi et al., 2019). The uncharacterized protein (C11orf84) or named SPIN1 docking protein (SPIN·DOC) would inhibit the expression of SPIN1 and the SPIN1-mediated Wnt signaling pathway (Bae et al., 2017). SPIN·DOC (rs643634) risk allele T carriers probably have lower gene expression of SPIN·DOC in GTEx eQTL result, which was compatible with our GWAS result. THOC5 regulates immediate-early gene response and contributes to the M-CSF-induced macrophage differentiation (Tran et al., 2013; Saran et al., 2013). In the absence of THOC5, mRNA export of M-CSF-inducible genes and regulators of myeloid differentiation were impaired. These data imply that THOC5 may have a role in macrophage differentiation (Tran et al., 2013). The effectively suppressed THOC5 expression resulted in a significant decrease of VSMC marker gene mRNA expression (Yuan et al., 2018). In our study, the carriers of THOC5 p.V525I (rs737976) allele C have high risk in ISR and have high mRNA expression which may lead to vascular smooth muscle cell migration and proliferation and ISR. GALNT2 promotes cell proliferation by activating the EGFR/PTEN-PI3K/Akt/mTOR signal pathway (Sun et al., 2019; Lin et al., 2014; Hu et al., 2018; Zhou et al., 2020). Active EGFR in vascular smooth muscle cells may facilitate chronic angiotensin II-induced arterial wall stiffening and media thickening (Schreier et al., 2018). SAMD11 was found to be widely expressed in many cell lines and ocular tissues, and its transcription was not regulated by CRX, OTX2, or NR2E3 proteins (Jin et al., 2013). Functional analysis indicated that human SAMD11 could promote cell proliferation (Jin et al., 2013). The prediction result of Polyphen2 HDIV showed that GALNT2 p.V516M (rs2273970) and SAMD11 p.H78Y (rs9988179) could change the structure and function of GALNT2 and SAMD11, respectively.

We successfully demonstrated the significant association between the five SNPs and new generation DES ISR. The DES ISR-GRS model could provide incremental biological information to interventional cardiologists prior to PCI and may be used to predict new generation DES ISR. This GRS model could be helpful for shared decision-making and could also remind interventionists to optimize PCI strategies in order to reduce ISR event rates.

We can make this score with a synergistic additive benefit with heart team discussion. If we calculate the DES-ISR GRS of the patients who have left main coronary artery disease or multi-vessel disease, this GRS could provide more information for the heart team to make the decision of PCI or CABG with the patients. Furthermore, when the patients have new generation DES ISR, we could also check their GRS and provide this genetic information to the interventionalist prior to decide the following treatment strategy: (1) PCI with drug-eluting balloon or another DES deployment for the ISR lesion or (2) referring to cardiovascular surgeons for bypass surgery. Currently, the DES-ISR SNP CHIP is still working and remains unavailable. However, when the commercial CHIP is available, we are able to check the GRS for those non-ST segment elevation patients with ACS who are eligible for PCI or CABG and get the data in a few minutes after coronary angiography. Then heart team members could discuss with the patients and their family to decide the best therapeutics.

### Limitations of the study

This study had several limitations. First, the sample size was small because the study was conducted at a single university hospital medical center, and the prevalence of new generation DES ISR was not high. Second, theoretically, Taiwan is a single nation-state/region. Our findings may not be applicable to other races. Third, we did not perform external validation to confirm our findings of the DES ISR-GRS model, although we did perform an internal validation. Fourth, we only focused on the genetic variants in exons and 3′-untranslated regions, not the whole genome. Fifth, we did not perform functional studies to investigate the biological impact of the SNPs evaluated in our GRS model. Therefore, our findings need to be tested in a larger prospective clinical trial with multiple races, and the biological consequences of these SNPs should be elucidated. Lastly, most of the enrolled patients were male. There may be some differences in the predictive power of the GRS related to sex. Thus, we cannot ensure that our DES ISR-GRS model could be used for estimating DES ISR probability in female patients.

## STAR★Methods

### Key resources table

**Table:** 

REAGENT or RESOURCE	SOURCE	IDENTIFIER
**Critical commercial assays**		

Infinium Asian Screening Array-24 v1.0 BeadChip	Illumina	CAT#20016318 https://www.illumina.com/products/by-type/microarray-kits/infinium-asian-screening.html
Infinium^Ⓡ^ HD Assay Kit WGS-PreMV1	Illumina	CAT#328735 https://www.illumina.com/products/by-type/accessory-products/infinium-hardware-kits.html
Infinium^Ⓡ^ LCG Assay Kit Post 1LMV2	Illumina	CAT#15043920 https://support.illumina.com/array/array_kits/humankaryomap-12-v1-beadchip-kit.html
Infinium^Ⓡ^ Assay Kit Post 2 LMV	Illumina	CAT#15023542 https://support.illumina.com/array/array_kits/humankaryomap-12-v1-beadchip-kit.html
Infinium^Ⓡ^ Assay Kit Post 4 LMV	Illumina	CAT#15043924 https://support.illumina.com/array/array_kits/humankaryomap-12-v1-beadchip-kit.html
Infinium^Ⓡ^ Assay Kit Single Post4 HV	Illumina	CAT#15023547 https://support.illumina.com/array/array_kits/humankaryomap-12-v1-beadchip-kit.html
QIAamp DNA Mini Kit	Qiagen	CAT#51304 https://www.qiagen.com/us/shop//sample-technologies/dna/qiaamp-dna-mini-kit/

**Deposited data**		

SNP statistic data	This study	https://github.com/dpc0628/ISR_2021

**Software and algorithms**		

base (R package)	The R Project for Statistical Computing, 2020	https://www.r-project.org
survminer (R package)	Kassambara et al., 2021	https://CRAN.R-project.org/package=survminer
Matchit (R package)	Ho et al., 2007	https://cran.r-project.org/web/packages/MatchIt/index.html
plink	Purcell et al., 2007	http://pngu.mgh.harvard.edu/purcell/plink/
Survival R code	This study	https://github.com/dpc0628/ISR_2021

### Resource availability

#### Lead contact

Further information and requests for resources and reagents should be directed to and will be fulfilled by the lead contact, Dr. Ping-Yen Liu (larry@mail.ncku.edu.tw).

#### Materials availability

This study did not generate new unique reagents.

### Experimental model and subject details

The clinical data and genetic profiles used in this study were obtained from National Cheng Kung University Hospital with IRB approval (A-ER-107-149), and it was registered to clinicaltrials.org (http://clinicaltrials.gov/ct2/show/NCT03877614). We provided the information including age, gender and sample size of the study in the Table 1.

### Method details

#### Study population

We recruited patients with CAD at National Cheng Kung University Hospital (NCKUH) from January 2010 to December 2019. The study protocol for clinical demographic data and genomic data collection was registered to clinicaltrials.org (http://clinicaltrials.gov/ct2/show/NCT03877614).(Hsu et al., 2020) Patients in the NCKUH CAD cohort who were greater than or equal to 20 years old and underwent next-generation DES insertion at de novo coronary artery stenotic lesions in the NCKUH cardiac catheterization lab were eligible for this study. Only those patients having the willingness to participate in this genomic research and providing the written informed consent were enrolled in this study. New generation DES were defined as the 2^nd^ generation DESs, including Xience (Abbott Vascular, Santa Clara, California) and Promus (Boston Scientific, Natick, Massachusetts) everolimus-eluting stents with durable polymer and the Resolute zotarolimus-eluting stents (Medtronic, Minneapolis, Minnesota) with durable polymer, and the 3^rd^ generation DESs: the Synergy everolimus-eluting stents (Boston Scientific, Natick, Massachusetts) with bioabsorbable polymer, the Biomatrix biolimus A9 (BA9)-eluting stents with biodegradable polymer and BioFreedom BA9-coated stents (Biosensors, Newport Beach, California), Nobori biolimus-eluting stents (Terumo, Tokyo, Japan) with biodegradable polymer, Ultimaster sirolimus-eluting stents (Terumo, Tokyo, Japan) with biodegradable polymer, and the Orsiro sirolimus-eluting stents (Biotronik, Bülach, Switzerland) with biodegradable polymer.

Because dialysis is a significant predictor of DES ISR (Shroff et al., 2013; Ikari et al., 2012; Ota et al., 2009; Otsuka et al., 2011; Shroff and Herzog, 2016), we excluded patients with chronic kidney disease stage 5 on dialysis (CKD 5D). Additionally, patients with missing clinical demographic data were also excluded. The written informed consent was obtained when the eligible patients agreed to participate in this study. We conducted this study according to the principles of the Declaration of Helsinki. The NCKUH Human Research and Ethics Committee approved this study (IRB: A-ER-107-149).

We recorded the demographic characteristics, comorbidities, medication history, PCI information, and new generation DES deployment data. ISR was defined as greater than or equal to 50% luminal narrowing of a stented coronary segment or within 5 mm of a stent edge at follow-up coronary arteriography (Dangas et al., 2010). All DES ISR events were confirmed by three qualified interventional cardiologists after comprehensively reviewing medical records and coronary angiographic images.

#### Derivation and validation cohorts

Six hundred thirty patients with CAD having new generation DES deployment were recruited in this study (Figure S1). Eligible patients who were retrospectively enrolled in this study (from January 2010 to December 2017) were assigned to the derivation cohort. From January 2018 to December 2019, we prospectively enrolled patients with CAD having DES deployment as the validation cohort.

#### Sample preparation for DNA extraction

We collected saliva samples from enrolled patients for DNA extraction (Mendoza et al., 2016). Patients were instructed to vigorously rinse their mouths for 30 s with 20 mL mouthwash (Listerine Cool Mint; Johnson & Johnson; 21.6% alcohol), and the mouthwash was then collected in a 50-mL tube. Once collected, saliva samples were stored at room temperature (approximate 20∼25°C) until use.

#### DNA extraction

To separate the mouthwash, the tubes were centrifuged at 2,000 × *g* for 10 min. We poured out the supernatant and added 200 μL phosphate-buffered saline to resuspend the cellular pellet. The resuspended solution was transferred into a new 1.5-mL tube. We added 200 μL buffer AL and 20 μL proteinase K (20 mg/mL) and then incubated the solution at 56°C for 10 min (QIAamp DNA Mini Kit; Qiagen, Valencia, CA, USA). Next, centrifugation was performed again to remove drops from the lid, and 200 μL ethanol was added for vortexing. We applied the mixture to a QIAamp spin column in a 2-mL collection tube and centrifuged the tube at 6,000 × *g* for 1 min. AW2 buffer (500 μL) was added to the QIAamp spin column in a clean 2-mL tube, which was centrifuged at full speed for 3 min. We eluted DNA with 30 μL Buffer AE into a clean 1.5-mL microfuge tube and then incubated the tube at room temperature for 1 min. Next, the tube was centrifuged at 6,000 × *g* for 1 min. We placed the tube at 4°C for short-term storage. The extracted DNA concentration was evaluated using a Qubit fluorometer.

#### Human genome-wide arrays

Genotyping was performed according to the Infinium Asian Screening Array (ASA)-24 v1.0 BeadChip (Illumina, Inc., San Diego, CA, USA) and standard Illumina protocols. The ASA aimed to capture coverage in Koreans, Mongolians, and Malaysians and was superior to existing reference populations, including populations of 2,000 Japanese, 1,600 Korean, hundreds of Taiwanese, 100 Malaysian, and 1000 Chinese (Mongolian and Singaporean) individuals. The clinical research content included variants associated with established cardiovascular diseases, relevant pharmacogenomics markers, and curated exonic content based on ClinVar, NHGRI, pharmacogenomic (PharmGKB), HLA variant, ACMG, and ExAC databases.

#### Genotyping quality control

Quality control was performed using PLINK 1.07 (http://pngu.mgh.harvard.edu/purcell/plink/) (Purcell et al., 2007). Samples were removed if any one of the following criteria was met: (1) per-individual call rate less than 97%; (2) per-individual autosome heterozygosity greater than 5 standard deviations from the mean; (3) wrongly assigned sex; (4) pihat greater than 0.2. Variants were removed if any one of the following criteria was met: (1) genotyping call rate less than 97%; (2) minor allele frequency less than 5%; (3) *p* value in Hardy-Weinberg equilibrium test less than 0.00001.

#### Next-generation DES ISR-GRS construction

We used the statistical software “R 3.4.0” with the “Matchit” package for propensity score matching and statistical analysis. The matching caliper was set to 0.2. After propensity score matching adjustment, there were 48 DES ISR (+) patients and 295 DES ISR (–) patients in the derivation cohort and 24 DES ISR (+) patients and 129 DES ISR (–) patients in the validation cohort.

We collected the recruited patients’ saliva and extracted DNA for GWASs. We focused on genetic variants in the exons and 3′-untranslated regions. The criteria for the candidate SNPs included (1) exonic SNPs, (2) SNP call rate greater than 0.97, (3) SNP allele frequency (AF) greater than 0.1, and (4) *p* value less than 0.005 (Figure 2 and Table S1). To select high-impact SNPs from these candidates, those meeting one of the following criteria would be selected for the DES ISR-GRS model: (1) gene expression in cardiovascular tissue or (2) possible functional impact of risk variants (defined as Poly-Phen 2 HDIV score > 0.446) (Table 2) (Adzhubei et al., 2010)^-28^. According to the number of risk alleles for each exonic SNP, values from 0 to 2 were assigned to each SNP. Then, all values for these candidate SNPs were summed up to generate a DES ISR-GRS.

### Quantification and statistical analysis

We expressed continuous data and dichotomous data as means ± standard deviations and numbers (percentages), respectively. Student’s *t*-tests were applied for comparison of normally distributed continuous variables, and nonparametric tests were used for comparison of continuous variables that were not normally distributed. Fisher’s exact tests were used for categorical variables. To identify significant variants between DES ISR (+) and DES ISR (–) patients in the derivation cohort, a logistic model was used to regress ISR status of variants, and the additive model was used to represent the variant genotype. Sensitivity, specificity, and accuracy were analyzed to determine the most optimal threshold of DES ISR-GRS. The Kaplan-Meier method was used with log-rank tests to compare DES-ISR free rates between strata. The Cox proportional hazards regression model was used to calculate hazard ratios (HRs). Results with two-tailed *p* values less than 0.05 were defined as statistically significant.

## Data Availability

The raw genetic data reported in this study cannot be deposited in a public repository because it was on a progressing clinic trial and for the data confidentiality. To request access, please contact Dr. Ping-Yen Liu, National Cheng Kung University Hospital. In addition, the statistic genetic data have been deposited at GitHub (https://github.com/dpc0628/ISR_2021) and are publicly available as of the date of publication. The uploaded files include the genotypes of 5 SNPs, and survival input data and code. All original code is available in this paper’s supplemental information and have been deposited at GitHub.
