# Supplementary material for: Genetic risk model for in-stent restenosis of second-and third-generation drug-eluting stents
Source: iScience. 2021 Sep 3;24(9):103082. doi: 10.1016/j.isci.2021.103082 (PMC8455661; doi:10.1016/j.isci.2021.103082)
Supplement: Document S1 — . Figures S1 and S2 and Tables S1–S3 [file mmc1.pdf]

iScience, Volume *24*

Supplemental information

Genetic risk model for in-stent restenosis of second-and third-generation drug-eluting stents

Yen-Wen Liu, Mu-Shiang Huang, Ling-Wei Hsu, Hsien-Yuan Chang, Cheng-Han Lee, Chi-Ying Lee, Dao-Peng Chen, Yi-Heng Li, Ting-Hsin Chao, Pei-Fang Su, Meng-Ru Shen, and Ping-Yen Liu

Figure S1. Algorithm for the genetic risk score (GRS) study of drug eluting stent (DES) in-stent restenosis (ISR), related to STAR method and Figure 1.

We included 2,749 patients receiving new-generation DES deployment in the National Cheng Kung University Hospital (NCKUH) coronary artery disease (CAD) cohort. Two hundred and five patients had DES ISR, which was confirmed by coronary angiography. After screening, only 690 CAD patients would like to participate in genomic research, and 92 patients had DES ISR. We excluded 60 patients, including 54 dialysis patients and 6 patients with missing clinical information. Finally, 630 patients were recruited for GRS analysis. These patients were categorized into 2 cohorts: derivation cohort and validation cohort.

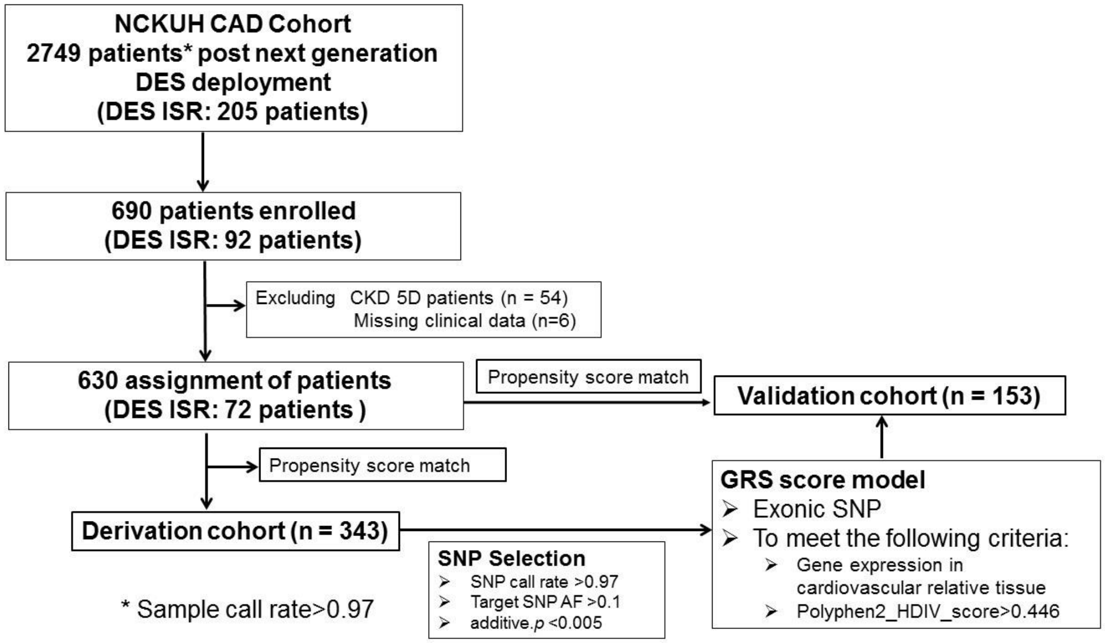

Figure S2. The possible mechanistical insight of the selected five single nucleotide polymorphisms (SNPs) for new generation drug eluting stent in-stent restenosis, related to Table 2.

Interaction of CAMLG with Angiotensin II type I receptor associated protein (ATRAP) may mediate the angiotensin II actions in vascular physiology. Spindlin1 (SPIN1) docking protein (SPINDOC) would inhibit the expression of SPIN1 and may result in cell proliferation. Moreover, the carriers of THOC5 p.V525I (rs737976) allele C have high risk in ISR and have higher mRNA expression, leading to vascular smooth muscle cell (VSMC) migration and proliferation and ISR. GALNT2 could activate the EGFR/PTEN-PI3K/Akt/mTOR signal pathway to promote VSMC proliferation (Sun et al., 2019, Lin et al., 2014, Hu et al., 2018, Zhou et al., 2020). The illustration was created with BioRender.com.

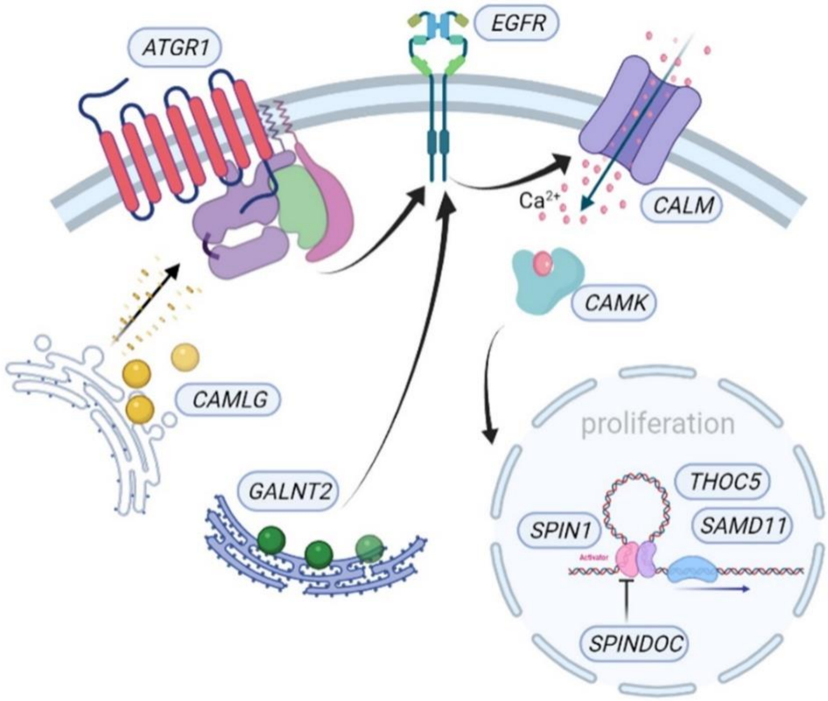

Table S1. Exonic single-nucleotide polymorphisms (SNPs) with significant difference between the patients with new-generation drug-eluting stent (DES) in-stent restenosis (ISR) and those patients without new-generation DES ISR, related to Figure 1.

rsID	p-value	Gene	GTEx_V6_tissue	Polyphen2_HDIV_score
rs12657663	0.0019	CAMLG	Artery	0
rs3802204	0.0006	DCSTAMP	Lung	0
rs16835683	0.0031	CSMD2	0	0
rs28687398	0.0038	LRRC2	0	0
rs11155787	0.0041	ZBTB2	0	0
rs11155787	0.0041	ZBTB2	0	0
rs1317421	0.0004	ENGASE	0	0
rs1805113	0.0004	TGFBR3	0	0
rs2147865	0.0044	GPR139	0	0
rs2273970	0.0024	GALNT2	0	0.764
rs2287866	0.0035	FCER2	0	0
rs2296354	0.0034	ARHGEF7	0	0
rs4804401	0.0013	OR7G2	Skin	0
rs643634	0.0047	C11orf84	Artery	0
rs737976	0.0012	THOC5	Artery	0
rs86487	0.0024	LARGE1	0	0
rs9988179	0.0021	SAMD11	0	0.989

Table S2. Genetic risk score distribution of patients with and without new-generation drug eluting stent (DES) in-stent restenosis (ISR) in derivation cohort, related to Figure 2.

Derivation cohort DES ISR genetic risk score	DES ISR (+) patient number	DES ISR (-) patient number	DES ISR (%)
0	1	17	5.56
1	2	67	2.90
2	7	92	7.07
3	11	71	13.41
4	12	34	26.09
5	12	13	48.00
6	1	1	50.00
7	2	0	100.00

Table S3. The overall predictive accuracy of different cutoff value of drug eluting stent (DES) in-stent restenosis (ISR) genetic risk score (GRS) in the derivation cohort, related to Figure 2.

Cutoff value of DES ISR GRS	Sensitivity (%)	Specificity (%)	PPV (%)	NPV (%)	Accuracy (%)
≥ 2	93.8	28.5	17.6	96.6	37.6
≥ 3	79.2	40.3	24.2	94.6	62.4
≥ 4	56.3	83.7	36.0	92.6	79.9

NPV, negative predictive value; PPV, positive predictive value.
